# 

**Published:** 1899-01-21

**Authors:** 


					>
ABSO????*URE. ^ ?? DRU?? OR0?" MK. CJSBD.
N^iy
JXO RA o
aoirVty?GT?AN Ihfi&uxm* _
013.
Vol. XXV. [aa*g Saturday,
Jan. 21st, 1899.
["ttM price twopjbjtce.

				

